# Anterior Ocular Biometrics as Measured by Ultrasound Biomicroscopy

**DOI:** 10.3390/healthcare10071188

**Published:** 2022-06-24

**Authors:** Mutasem Elfalah, Mona Mohammad, Mario Damiano Toro, Nakhleh Abu-Yaghi, Robert Rejdak, Yacoub A. Yousef

**Affiliations:** 1 School of Medicine, The University of Jordan, Amman 11941, Jordan; m.alrabie@ju.edu.jo (M.E.); n.abuyaghi@ju.edu.jo (N.A.-Y.); 2 Department of Surgery (Ophthalmology), King Hussein Cancer Centre, Amman 11941, Jordan; dm.11804@khcc.jo; 3 Chair and Department of General and Pediatric Ophthalmology, Medical University of Lublin, 20-079 Lublin, Poland; robert.rejdak@umlub.pl; 4 Eye Clinic, Public Health Department, University of Naples Federico II, 80131 Naples, Italy

**Keywords:** anterior segment, ultrasound biomicroscopy, iris, ciliary body

## Abstract

Background: High frequency ultrasonography (ultrasound biomicroscopy; UBM) is an ophthalmic diagnostic tool that can be used to measure the depth of the anterior segment (ASD), the anterior chamber angle (ACA), as well as thicknesses of the iris and the ciliary body (CB). Methods: The anterior segment dimensions and thicknesses were measured by Sonomed 35-MHz. Results: Measurements for 95 eyes from 52 adults were analyzed. The mean and median ASD and ACA were 2.91, 2.92 ± 0.41 mm and 34.1, 34.3 ± 12.1 degrees, respectively. The angle superiorly was wider than inferiorly (*p* = 0.04). At the root of the iris, the mid of the iris, and the juxtapupillary edge of the iris, the iris thicknesses (median, mean) were 0.40, 0.41 ± 0.1, 0.50, 0.51 ± 0.1, and 0.70, 0.71 ± 0.1 mm, respectively. The thicknesses of CB and CB together with the ciliary processes (median, mean), were 0.70, 0.71 ± 0.15 mm and 1.36, 1.41 ± 0.15 mm, respectively. The upper quadrant of both the iris and the CB was significantly thicker than the lower quadrant (*p* = 0.04). Conclusions: Our biometric measurements for the anterior segment can be used as normative data for anterior segment depth and angle and iris and ciliary body thickness in normal eyes.

## 1. Introduction

Various ophthalmic pathologies have the potential to lead to structural changes in the anterior ocular components such as the anterior segment depth (ASD, anterior chamber angle (ACA), and the ciliary body (CB) and iris 1–3. Diagnostic modalities to pick up such changes include high-frequency ultrasonography (ultrasound biomicroscopy; UBM) and optical coherence tomography (OCT). Examples are structural misalignment caused by ocular tumors (e.g., uveal melanoma (iris or ciliary body), iris metastasis, ciliary body leiomyoma, and others) [1,2,3,4,5], or abnormal thickening caused by infiltration (e.g., uveitis, metastasis, lymphoma, metastasis, granulomas, and other infiltrative tumors) [6,7,8,9]. Other recognized causes of anterior segment morphology alterations are glaucoma and intraocular implants [10,11,12,13].

Since its development in the 1990s, UBM has been utilized to obtain microscopic high-resolution sections of the anterior compartment of the globe. In contrast to regular ultrasound techniques (B-scan or A-scan, which operate with a frequency of 8–12 MHz), UBM operates at a frequency of 36–100 MHz 3–7. This yields axial resolution of up to 20um, lateral resolution of up to 50 um, and 4–5 mm tissue penetration. Using high-frequency ultrasound biomicroscopy (UBM), the anterior portion of the eye may be seen. The depth of the anterior segment (ASD), the angle of the anterior chamber (ACA), and the thickness of the iris and ciliary body may all be measured using this method. As a result, it is used to diagnose a variety of anterior chamber conditions, including congenital anterior chamber abnormalities, open-angle glaucoma, cataracts, iris problems, iris, and ciliary cancers, and various conjunctival infections [3,4,5,6,7,8,9,10,11].

Quantitative data on the anatomy of the anterior compartment of the eye (such as ASD, ACA, and iris and ciliary body thickness) is important for surgical planning for many ocular diseases [14], and it is also required as comparative normal data when evaluating multilocular disease. The information in the literature is scarce regarding the typical measurements (biometrics) of the normal anterior segment of the eye, this was assessed by very few UBM and anterior segment OCT studies [13,15,16]. Therefore, we tried here to measure the anterior biometrics for normal eyes for a population in our community. This was performed using ultrasound biomicroscopy by the same operator who then measured the most important anterior segment biometics including ASD, ACA, iris thickness, ciliary body thickness, and sulcus-to-sulcus distance (STS), and these data were analyzed by looking into subgroups to see the differences based on sex, age, height, and side of the eye. This study evaluated a homogeneous Middle Eastern population to add to the literature’s limited existing data on normative anterior segment biometrics. Our results are expected to help ophthalmologists in evaluating the eyes with anterior segment diseases for diagnosis and surgical planning.

## 2. Materials and Methods

A retrospective analysis of 95 eyes from 52 individuals was conducted. These individuals underwent ophthalmic examination including UBM and were found to have no ocular structural abnormalities, in the period from 2015 to 2020. Patients were included if access to their medical records and UBM images was available. Patients were assessed concerning age, sex, eye side, weight, and height. Approval of the KHCC institutional review board was obtained (20 KHCC 127). Thirty-five MHz UBM images were taken at four consecutive sites (3, 6, 9, and 12 o’clock). Consent was obtained before each test.

### 2.1. Inclusion and Exclusion Criteria

UBM is part of the routine workup for patients with various types of malignancies at KHCC to exclude intraocular malignancy or metastasis or to compare the measurement of an eye affected by a tumor such as ciliary body ring melanoma, to those of the unaffected eye. Eyes were included in the study if the ophthalmological examination was normal, with no or mild refraction error (spherical error between −2.0 and +2.0), the examination was performed by Y.A.-Y., examination involved four standard sites (3, 6, 9, and 12 o’clock), and access to documented results was available.

Eyes were excluded if any pathology was detected on examination, or if the patient had glaucoma, previous ocular trauma, history of invasive intraocular surgery, high refractive error, or previous radiotherapy to the eye or the head and neck.

### 2.2. Examination Technique

**Figure 1:**
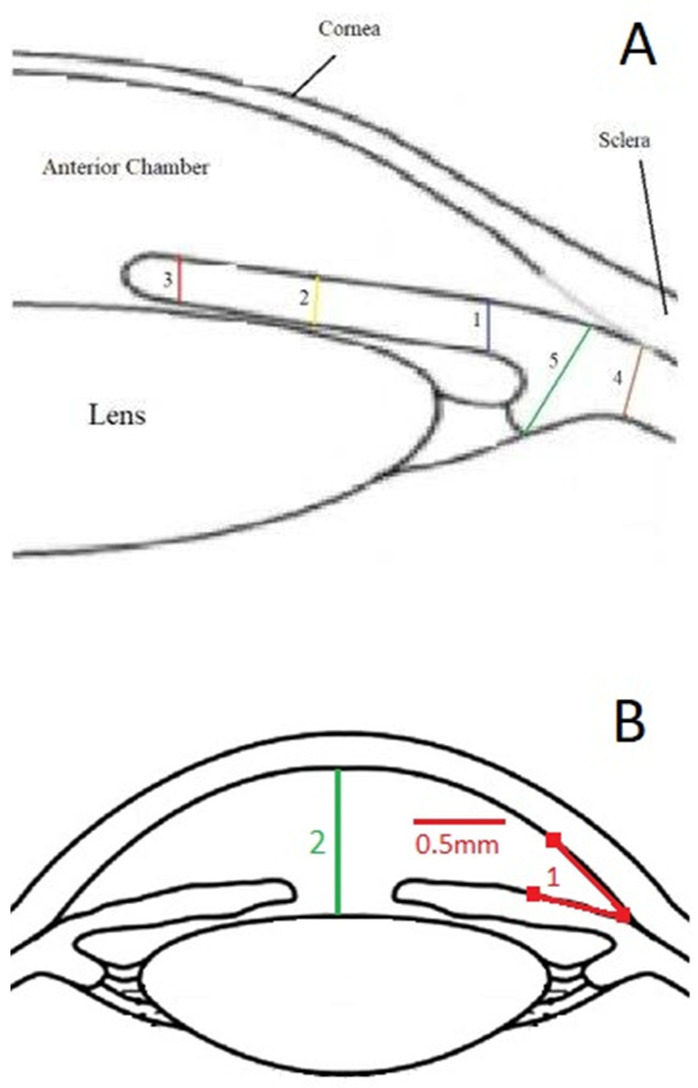
(**A**) This is an illustration of both the iris and the ciliary body, as seen by ultrasound biomicroscopy (UBM) in this study. The blue line (1) represents the iris thicknesses at 0.8 mm distance from the root of the iris. The yellow line (2) represents the iris thickness halfway down the radial length of the iris. The red line (3) represents the iris thickness at the juxtapupillary edge. The blue line (4) represents the thickness of the ciliary body, and the green line (5) represents the thickness of the ciliary body and the ciliary processes. (**B**) The red line (1) represents the anterior chamber angle, and the green line (2) represents the anterior segment depth, all as described in the methodology section.

Examinations were conducted at the outpatient eye clinic with the patient supine facing the ceiling. The transducer was directed and the probe was manipulated by the image on the screen. Topical anesthesia (Oxybuprocaine hydrochloride 0.4%) was applied. The eyelids were separated and a water bath was made with a silicon immersion eye cup of different sizes (large 24 mm, medium 22 mm, and small 20 mm) filled with distilled water or normal saline and pushed on the eye to prevent fluid leakage. To keep fluid from seeping out of the eye, it was squeezed against it. The 35-MHz high-frequency ultrasonography equipment (SONOMED, Inc., New York, NY, USA) was employed to aquire radial (longitudinal) images of the anterior compartment of the eye at the three, six, nine, and twelve o’clock locations of each eye. Images were analyzed retrospectively, and the required readings were obtained by utilizing the integrated measurement calipers in the system (See Figure 1A,B):

1. The anterior chamber angle (in degrees) was calculated using two 0.5 mm lines that began at the iris root and traveled across the posterior cornea and the anterior surface of the iris (Figure 1B). Images were obtained from each quadrant (upper, lower, nasal, and temporal).
2. To determine the anterior segment depth (ASD), the axial distance between the anterior corneal surface and the anterior surface of the lens was measured; views were as vertical as feasible, as indicated on the screen (See Figure 1B).
3. The thicknesses of the iris were measured at 0.8 mm from the iris root, amid the iris, and at the juxtapupillary edge of the iris (thickest area of the iris at 1 mm distance from the pupil).
4. The perpendicular distance between the apex of the ciliary body (inner tip) and the inner wall of the sclera was measured to assess CB thickness. The distance between the inner-most point of the ciliary process and the inner wall of the sclera was used to calculate the CB + ciliary process thickness (perpendicular to the scleral wall).
5. The sulcus-to-sulcus diameters (STS) were measured as cross-sectional images and were obtained on the following two meridians: vertical (up-down, 90°) and horizontal (nasal-temporal 180°). The STS diameters were measured offline in the images with the widest pupil diameter.

**Figure 2:**
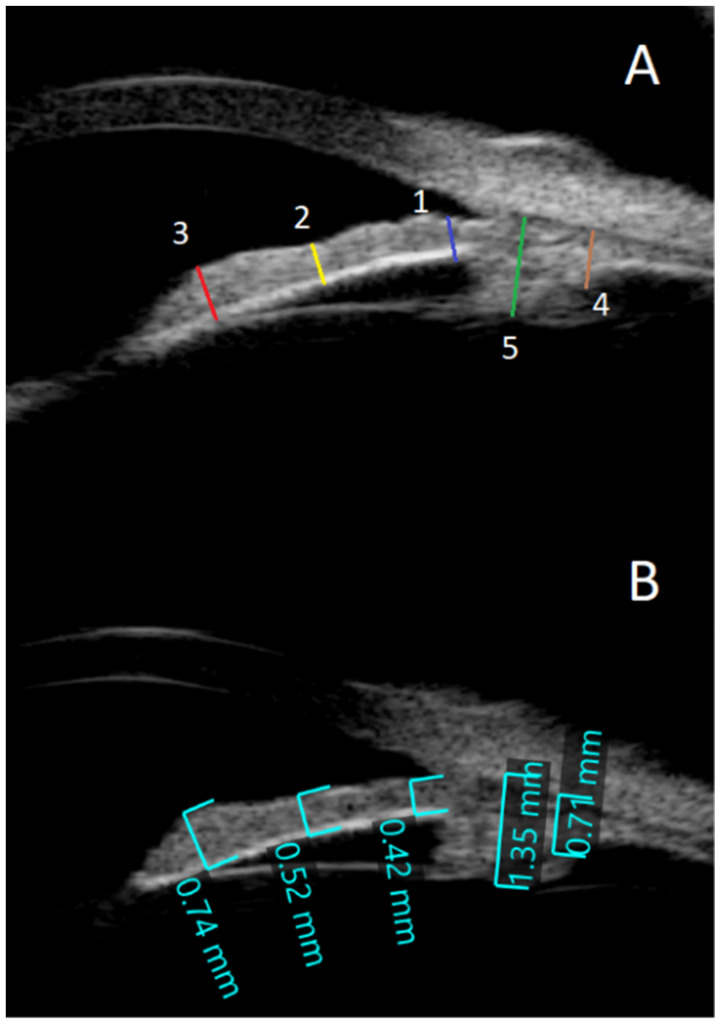
Ultrasound biomicroscopy (UBM): (**A**) This is an example of the UBM that shows the measured thicknesses for the iris and the ciliary body at different locations. The blue line (1) represents the iris thicknesses at 0.8 mm distance from the root of the iris. The yellow line (2) represents the iris thickness halfway down the radial length of the iris. The red line (3) represents the iris thickness at the juxtapupillary edge. The blue line (4) represents the thickness of the ciliary body, and the green line (5) represents the thickness of the ciliary body and the ciliary processes. (**B**) The iris thickness of one of the participants was 0.42 mm at a distance of 0.8 mm from the iris root, 0.52 mm in the mid of the radial length of the iris, and 0.7 mm at the juxtapupillary edge. The ciliary body was 0.72 mm thick, while the ciliary body with ciliary processes was 1.38 mm thick.

Only one person (Y.A.Y.) was responsible for obtaining all images and measurements. The readings were obtained in a sun-lit room with the iris in a normal physiological position. To prevent any change in the axial length or thickness of the iris due to mydriasis, dilating drops were avoided. Each measurement was obtained twice, and the average of the two readings was used in this study (Figure 2).

### 2.3. Statistical Analysis

In this study, the mean and median thicknesses, range, and standard deviation were all used as statistical factors. The statistical difference in means between the different groups was determined using a *t*-test. A *p*-value of 0.05 or less is significant.

## 3. Results

The participants in the research consisted of adult patients of Middle Eastern ethnicity. All patients had brown eyes. There were 40% female and 60% male, with an average age of 39 (median 38 years, range 22–68).

### 3.1. Anterior Segment Depth and Anterior Chamber Angle Measurements

The mean and median depths of the anterior segments were 2.92 and 2.91 ± 0.41 mm, respectively (range: 2.51 to 3.33 mm); while the mean and median anterior chamber angle was 34.3 and 34.1 ± 12.1 degrees (range: 22.2 to 46.4 degrees). Age, sex, eye side, or patient height had no statistically significant effect on ASD or ACA (Table 1). The nasal and temporal quadrants did not differ (*p* = 0.86); however, the upper quadrant had a wider anterior chamber angle than the lower quadrant (*p* = 0.04) (Table 1). The mean and median vertical and horizontal STS were 12.48, 12.49 ± 1.70 mm and 12.22, 12.24 ± 1.60 mm, respectively.

### 3.2. Iris Measurements

The measured irises’ thickness (mean and median) was 0.41, 0.40 ± 0.1 mm (range: 0.28 to 0.65 mm) at 0.8 mm from the root of the iris, 0.51, 0.50 ± 0.1 mm (range: 0.30 to 0.73 mm) at mid of the irises’ radial length, and 0.71, 0.70 ± 0.1 mm at the irises’ thickest area near the juxtapupillary edge (range: 0.41 to 0.93 mm). Iris thickness was not affected by age, sex, eye side, or patient height in a statistically significant way. The nasal and upper quadrants were substantially thicker than the temporal and lower quadrants (*p* = 0.04), respectively (Table 2).

### 3.3. Ciliary Body Measurements

The measured ciliary body thickness (mean and median) were 0.71 and 0.70±0.15 mm, respectively (range: 0.42 to 1.14 mm); while the CB + ciliary processes were 1.41 and 1.36 ± 0.15 mm, respectively (range: 1.19 to 1.75 mm). The measured ciliary body thickness (mean and median) were 0.71 and 0.70 ± 0.15 mm, respectively (range: 0.42 to 1.14 mm); while the CB + ciliary processes were 1.41 and 1.36 ± 0.15 mm, respectively (range: 1.19 to 1.75 mm). CB thickness and CB + ciliary processes thickness were similar between male and female participants, and between the right eyes and the left eyes (*p* = 0.68 and *p* = 0.48, respectively).

It was noted that individuals below 40 years of age, and those taller than 160 cm were more likely to have thicker CB + ciliary processes; however, this did not reach statistical significance (Table 3). The nasal and temporal eye quadrants did not differ substantially in measurements (*p* = 0.8); however, the upper quadrants were significantly thicker than the lower quadrants (*p* = 0.04) (Table 3).

## 4. Discussion

With diverse ocular diseases, the anatomy of the anterior segment of the globe (including ASD and ACA), as well as the shape and thickness of the anterior uveal tissue (the iris and the ciliary body), changes. Therefore, normative biometrics data about anterior segment parameters of the eye (including ASD and ACA) as well as normative data about iris thickness and ciliary body thickness, are important as comparative biometrics measures to evaluate ocular abnormalities based on normal reference biometrics. Furthermore, high-frequency ultrasound biomicroscopy can define normal vs. abnormal anterior segment biometrics quantitatively.

Ultrasound biomicroscopy is an important technique that can study multiple pathologies that affect the architecture of the anterior segment of the eye globe, including cataracts, glaucoma, and congenital defects. These scans may have an impact on preoperative planning depending on the condition of each eye. To distinguish between normal and pathological UBM findings, knowledge of expected normal parameters and biometrics of the anterior segment of the eye globe are needed [14]. UBM has also been shown to be a beneficial diagnostic technique for diagnosing the cause of pseudophakic glaucoma, since it allows for clear viewing of distinct angle structures [17]. Secondary angle closure, induced by anterior synechiae development, is one of the most common causes of post-penetrating keratoplasty glaucoma in eyes with opaque media, which should be checked clinically before surgery by UBM to prevent such difficulties and help predict the procedure’s outcome [18]. In such circumstances, UBM can be a helpful tool for anterior-segment assessment and can aid in the planning of glaucoma filtering procedures and drainage devices. It is for this reason that normative UBM data are required.

The mean anterior segment depth was found to be about 2.42 ± 0.34 mm in an OCT investigation of 2985 participants. Female sex, age, hyperopia, nuclear cataract, short body height, and angle-closure glaucoma were all found to be associated with shallow anterior segment depth [19]. In this study, the mean and median anterior segment depth was 2.92 ± 0.41 mm, which is slightly wider than that report. This is because we included only normal eyes and we excluded all eye pathologies (that may be associated with shallow AC) such as cataract, hyperopia, and glaucoma. There were no differences in the measured anterior segment depth based on sex, age, patient height, or eye side.

Patients over 60 exhibited a 0.2 mm shorter anterior chamber depth and a 1.0-degree narrower anterior chamber angle than those under 40, despite no significant differences in ASD or ACA across age groups. Furthermore, other reports found that the older cohort’s ACA was 4.5 degrees smaller on average than the younger cohort’s, and that age was related to shallow anterior segment depth [19,20,21,22,23]. The constant development and thickening of the lens throughout life might explain these observations [24]. Furthermore, Friedman et al., showed that in the supine position, the UBM findings showed that the inferior angle is narrower than the superior angle 11, which is similar to our findings where the lower ACA was significantly narrower than the upper angle. Of interest, the mean vertical STS was 12.48 mm, which is higher than horizontal STS, which was 12.22 mm, and both were equal or slightly larger than the mean vertical (12.46 mm) and horizontal (11.9 mm) STS measured in China [25].

A study performed on a patient sample in New York reported measurements of the iris and ciliary body that are comparable to the outcomes in this research [12]; however, the Jordanian patient sample demonstrated slightly thicker iris and ciliary bodies. A study of [26] Japanese control patients revealed slightly less CB thickness than results from New York and Jordan [12].

This study population demonstrated a large range of the measured iris and CB + ciliary process thickness. Several variables could potentially influence this, such as iris color, pupil size, and physiological changes of the pupil size during accommodation [15,16,27,28,29,30]. The use of dilating eye drops is another possible factor; however, the use of pharmacological dilatation was avoided in this study, to eliminate this effect. This study also explores other variants including age, height, and ethnicity, in addition to ocular pathology or surgery. Eye pathology and surgery were excluded from our study. Physiological changes due to accommodation to changes in the light conditions during the examination, however, are difficult to rule out. Similarly, Garcia et al., found a wide range of iris thickness measurements (0.2–0.9 mm) despite avoiding pharmacological dilatation [12]. Pupil size could not be accurately estimated or evaluated in this study as there was noticeable variation throughout the examination. All patients in this sample had brown iris, this is not sufficient, however, to explain the difference in thickness between our study and the New York patient sample, as all but two of the latter also had brown eyes. Ethnicity does not explain the variation in measurements between different patients in this study, as all participants were of Middle Eastern descent. However, larger-scale studies are required to decide if the ethnic origin is a determinant of CB thickness.

The findings of this study demonstrated that height and sex were not significant determinants of iris and CB thickness. Henzan et al., studied normal globes in patients aged 40 and above in the population-based Kumejima study, and demonstrated age-related thinning of the iris [16]. A similar trend was found in our study, but it lacked statistical significance. Variation in iris thickness may merely represent normal variation between individuals.

Another technique for obtaining high-resolution cross-sectional pictures utilizing multiple wavelengths of light is anterior segment OCT imaging (830–870 nm and 1310 nm) [26,27,30]. Compared to the OCT, UBM can be used to produce images of posterior structures such as the ciliary body, zonules, and peripheral lens, but it causes more discomfort during the examination and is operator dependent. Both modalities can be utilized to obtain real-time images of the anterior segment [1,3,12,26,27]. The mean iris thickness that was assessed using OCT at two locations (750 nm and 2,000 nm from the scleral spur) was 0.406 mm and 0.514 mm, respectively [28]. We opted for UBM in this research as it offers superior penetration and details of the posterior structures [29].

Normal data (biometrics) about the anterior component of the eye globe including anterior segment depth, ACA, iris thickness, and ciliary body thickness are needed before deciding if this eye is normal or abnormal, and these data are missing in the literature [31]. These structures can be evaluated by either UBM or anterior segment OCT [32,33], and differences may exist between different populations [34,35,36], and may exist with special ocular diseases including intraocular tumors, glaucoma, myopia, and retinal detachment [37,38,39,40].

Using high-frequency ultrasound biomicroscopy, we attempted to quantify the normal anterior segment biometrics such as anterior chamber depth, anterior chamber angle, CB thickness, and iris thickness in normal eyes from a homogeneous population in Jordan. We followed the same strict procedure that we use when we image the eyes as needed at a tertiary cancer center. Therefore, our results of biometrics on normal eyes are repeatable in clinical practice, our eye-to-eye variations in measured biometrics are part of normal variation in the population, and the underlying causes behind these variations are not well determined yet.

Our findings are important as they represent homogenous eyes from adults of the same ethnicity, even though it is a retrospective study, of a limited number of eyes for 46 adults. We believe our results should be a good additional reference for ophthalmologists, specifically in the Middle East, until larger more comprehensive prospective studies are performed and identify the normal anterior segment biometrics and the significant variables that may affect them.

## 5. Conclusions

In conclusion, the superior ACA was wider than the inferior angle, the nasal and upper iris thicknesses were greater than the temporal and lower iris thicknesses, and the thickness of the upper CB + ciliary processes was greater than the lower thickness. The biometrics of the anterior segment, as well as the thickness of the iris and CB, were unaffected by age, sex, eye side, or patient’s height. The findings of this study can be used as normative data for biometrics of the anterior segment.

## Figures and Tables

**Table 1:** The impact of demographics, eye side, and the quadrant of the eye on the anterior chamber angle (ACA) and the anterior segment depth (ASD) in 95 normal eyes.

Parameter	Anterior Chamber Depth (mm)	Anterior Chamber Angle (Degree)
Overall (95 eyes)	2.92,2.91 ± 0.41	34.3, 34.1 ± 12.1
Sex		
Female (n = 37 eyes, 21 patient)	2.90,2.90 ± 0.40	34.1, 34.0 ± 12.1
Male (n = 58 eyes, 31 patient)	2.96,2.93 ± 0.41	34.4, 34.2 ± 12.1
*p*-value	0.58	0.52
Age (year)		
20–40 (50 eyes)	3.01,2.92 ± 0.41	34.8, 34.6 ± 12.1
40–60 (29 eyes)	2.91,2.91 ± 0.40	34.3, 34.2 ± 12.1
60–80 (10 eyes)	2.88,2.90 ± 0.39	33.9, 33.8 ± 12.1
*p*-value	0.24	0.18
Height		
Less than 160 cm (55 eyes)	2.91,2.90 ± 0.38	32.2, 32.1 ± 12.2
More than 160 cm (40 eyes)	2.94,2.92 ± 0.41	35.3, 34.1 ± 12.1
*p*-value	0.78	0.42
Eye		
OD (46 eyes)	2.93,2.92 ± 0.41	34.4, 34.2 ± 12.1
OS (49 eyes)	2.91,2.90 ± 0.41	34.3, 34.1 ± 12.1
*p*-value	0.89	0.91
Quadrant		
Upper quadrant (95 eyes)	NA	35.2, 34.2 ± 12.1
Lower quadrant (95 eyes)	NA	33.8, 34.1 ± 12.1
*p*-value		0.04
Nasal quadrant (95 eyes)	NA	34.5, 34.3 ± 12.1
Temporal quadrant (95 eyes)	NA	34.2, 34.0 ± 12.1
*p*-value		0.86

SD = standard deviation.

**Table 2:** Iris thickness (mm) comparison by sex, age, height, eye and quadrant.

Parameter	Iris Root Thickness * [Mean, Median ± SD (range)]	Mid of the Iris [Mean, Median ± SD (Range)]	Tip of the Iris [Mean, Median ± SD (Range)]
Measured thickness of the iris (95 eyes)	0.41, 0.40 ± 0.1 (0.28–0.65)	0.51, 0.50 ± 0.1 (0.30–0.73)	0.71, 0.70 ± 0.1 (0.41–0.93)
Sex (Male/Female)			
Female (37 eyes, 21 patients)	0.40, 0.40 ± 0.1 (0.28–0.62)	0.50, 0.50 ± 0.1 (0.30–0.70)	0.70, 0.69 ± 0.1 (0.41–0.88)
Male (58 eyes, 31 patients)	0.41, 0.40 ± 0.1 (0.29–0.65)	0.51, 0.50 ± 0.1 (0.31–0.73)	0.72, 0.71 ± 0.1 (0.43–0.93)
*p*-value	0.89	0.89	0.82
Age (in years)			
Less than 40 (44 eyes)	0.41, 0.40 ± 0.1 (0.29–0.65)	0.51, 0.50 ± 0.1 (0.32–0.73)	0.71, 0.70 ± 0.1 (0.42–0.93)
More than 40 (41 eyes)	0.41, 0.40 ± 0.1 (0.28–0.62)	0.50, 0.50 ± 0.1 (0.30–0.70)	0.70, 0.70 ± 0.1 (0.41–0.90)
*p*-value	1.00	0.89	0.93
Height (in cm)			
Less than 160 cm (55 eyes)	0.40, 0.40 ± 0. 0.1 (0.29–0.61)	0.51, 0.50 ± 0.1 (0.31-0.70)	0.71, 0.70 ± 0.1 (0.41–0.91)
More than 160 cm (40 eyes)	0.41, 0.40 ± 0.1 (0.28–0.65)	0.51, 0.50 ± 0.1 (0.32–0.73)	0.72, 0.70 ± 0.1 (0.41–0.93)
*p*-value	0.84	0.84	0.93
Side			
OD (46 eyes)	0.40, 0.39 ± 0.1 (0.32–0.65)	0.50, 0.49 ± 0.1 (0.30–0.73)	0.70, 0.69 ± 0.1 (0.41–0.93)
OS (49 eyes)	0.39, 0.39 ± 0.1 (0.28–0.64)	0.50, 0.50 ± 0.1 (0.30–0.72)	0.70, 0.69 ± 0.1 (0.41–0.91)
*p*-value	0.93	0.94	0.89
Measured Quadrant of the Eye			
Upper quadrant (95 eyes)	0.43, 0.41 ± 0.1 (0.30–0.63)	0.53, 0.51 ± 0.1 (0.30–0.70)	0.74, 0.73 ± 0.1 (0.44–0.91)
Lower quadrant (95 eyes)	0.37, 0.36 ± 0.1 (0.28–0.62)	0.47, 0.46 ± 0.1 (0.31–0.71)	0.67, 0.66 ± 0.1 (0.41–0.89)
*p*-value	0.045	0.043	0.018
Nasal quadrant (95 eyes)	0.43, 0.41 ± 0.1 (0.30–0.65)	0.53, 0.51 ± 0.1 (0.32–0.73)	0.73, 0.71 ± 0.1 (0.47–0.93)
Temporal quadrant (95 eyes)	0.40, 0.39 ± 0.1 (0.28–0.62)	0.48, 0.47 ± 0.1 (0.30–0.70)	0.69, 0.69 ± 0.1 (0.41–0.90)
*p*-value	0.046	0.040	0.016

SD = standard deviation. * This was taken at a distance of 0.8 mm from the root of the iris.

**Table 3:** Ciliary body thickness (mm) comparison by sex, age, height, eye, and quadrant.

Parameter	The Thickness of the Ciliary Body [Mean, Median ± SD (Range)]	The Thickness of the Ciliary Body + Ciliary Processes [Mean, Median ± SD (Range)]
Measured thickness (83 eyes)	0.71, 0.70 ± 0.15 (0.42–1.14)	1.41, 1.36 ± 0.15 (1.19–1.75)
Sex		
Female (n = 37 eyes, 21 patients)	0.69, 0.68 ± 0.15 (0.42–1.14)	1.39, 1.34 ± 0.15 (1.19–1.70)
Male (n = 58 eyes, 31 patients)	0.71, 0.69 ± 0.15 (0.44–1.12)	1.43, 1.37 ± 0.15 (1.31–1.75)
*p*-value	0.34	0.44
Age (year)		
Less than 40 (44 eyes)	0.74, 0.73 ± 0.15 (0.46–1.14)	1.43, 1.39 ± 0.15 (1.24–1.75)
More than 40 (41 eyes)	0.69, 0.68 ± 0.15 (0.42–1.08)	1.37, 1.35 ± 0.15 (1.19–1.60)
*p*-value	0.56	0.68
Height		
Less than 160 cm (55 eyes)	0.70, 0.69 ± 0.1 (0.42–1.1)	1.39, 1.33 ± 0.1 (1.19–1.60)
More than 160 cm (40 eyes)	0.73, 0.71 ± 0.2 (0.45–1.14)	1.44, 1.37 ± 0.2 (1.29–1.75)
*p*-value	0.92	0.76
Eye		
OD (46 eyes)	0.71, 0.70 ± 0.15 (0.43–1.12)	1.42, 1.37 ± 0.15 (1.29–1.75)
OS (49 eyes)	0.71, 0.69 ± 0.15 (0.42–1.14)	1.40, 1.35 ± 0.15 (1.19–1.68)
*p*-value	0.68	0.48
Measured site (Quadrant)		
Upper quadrant (95 eyes)	0.73, 0.71 ± 0.15 (0.45–1.12)	1.44, 1.36 ± 0.15 (1.33–1.75)
Lower quadrant (95 eyes)	0.69, 0.68 ± 0.15 (0.42–1.10)	1.39, 1.34 ± 0.15 (1.19–1.67)
*p*-value	0.044	0.031
Nasal quadrant (95 eyes)	0.72, 0.71 ± 0.15 (0.45–1.12)	1.43, 1.37 ± 0.15 (1.33–1.75)
Temporal quadrant (95 eyes)	0.69, 0.688 ± 0.15 (0.42–1.10)	1.39, 1.34 ± 0.15 (1.19–1.67)
*p*-value	0.27	0.88

SD = standard deviation.

## Data Availability

Data are available on reasonable request upon demand to the corresponding authors.
